
JOURNAL INFORMATION
==============================
NLM Title Abbreviation: ScientificWorldJournal
Journal ID: ScientificWorldJournal
NLM Journal ID: 101131163
Journal ID: TSWJ

The Scientific World Journal

ISSN: 2356-6140
EISSN: 1537-744X
Publisher: Wiley

ARTICLE INFORMATION
==============================
PMCID: PMC5900487
PMID: 17891312
DOI: 10.1100/tsw.2007.194
Article ID: 709174
Article version: 1
Subjects: Editorial

Spina Bifida Advocacy – A Case Study In Public Policy Success

Brownstein Cindy 1 *
Paul Ilisa Halpern 2
Quinn Anne House 3
1 Spina Bifida Association, Washington, DC, USA
2 Drinkler Biddle Gardener Canton, Washington, DC, USA
3 Quinn Marketing & Development, Inc., Washington, DC, USA
*Corresponding author.Academic Editor: David B. Joseph

Publication date: 2007
Electronic publication date: 2007 Sep 17
Volume: 7
First page: 1538
Last page: 1543
Received 2007 Jul 6; Accepted 2007 Jul 6
Copyright: Copyright © 2007 Cindy Brownstein et al.
Copyright year: 2007
License: This is an open access article distributed under the Creative Commons Attribution License, which permits unrestricted use, distribution, and reproduction in any medium, provided the original work is properly cited.
License URL: https://creativecommons.org/licenses/by/3.0/

==============================
Editorial
Special Issue: Challenges in Spina Bifida
*TheScientificWorldJOURNAL*, (2007) **7**, 1538–1543
TSW Urology
ISSN 1537-744X; DOI 10.1100/tsw.2007.194

TheScientificWorldJOURNAL
www.thescientificworld.com

### Spina Bifida Advocacy – A Case Study In Public Policy Success

Cindy Brownstein[1*], Ilisa Halpern Paul[2], and Anne House Quinn[3]
*[1]Spina Bifida Association; [2]Drinkler Biddle Gardener Canton; [3]Quinn Marketing & Development, Inc., Washington, DC*

*Received July 6, 2007; Accepted July 6, 2007; Published September 17, 2007*

---

### INTRODUCTION

Advocacy. Public policy. Politics. Congress and the Administration. Given recent events, these words often cause people to groan, roll their eyes, tune-out, or launch into a litany of complaints about the government. While each of these responses is understandable, the Spina Bifida Association (SBA) maintains a steadfast commitment to working with individuals, families, physicians, nurses, and other caregivers to channel their sense of frustration with government, public policy, and regulations into positive change for the Spina Bifida community. SBA engages in health policy advocacy to help fulfill its mission and achieve its goals and objectives as an organization to enhance the lives of those with Spina Bifida and those whose lives are touched by this challenging birth defect. To that end, SBA seeks local, state, federal support for policies and programs that reduce and prevent suffering from Spina Bifida.

Each year, SBA establishes its Public Policy Agenda by taking into consideration a number of factors, including: the SBA mission, SBA goals and objectives, previous SBA public policy priorities and positions, the current political climate at the national level, and whether or not SBA has a particular or unique role to play with regard to a specific issue or matter. Principally, for the past four years, SBA's advocacy efforts have focused on increasing funding for the National Spina Bifida Program housed at the U.S. Centers for Disease Control and Prevention (CDC).

### Why and How the Spina Bifida Community Engages in Advocacy

For better for worse, health care is a political issue and one that the government funds, debates, and regulates. As such, unless policymakers hear from members of the Spina Bifida community about how proposed funding levels, policies, and programs will affect them (positively or negatively), they will make decisions without full understanding of the impact of their choices. Most Members of Congress and their staffers do not know anyone who has Spina Bifida and as such, they have limited understanding and appreciation of the challenges of living with this birth defect. Therefore, it is imperative for affected individuals and family members as well as caregivers to (1) help educate elected officials and their staffers about the needs of individuals with Spina Bifida and (2) advocate funding, policies, and programs that will improve quality of life for the 70,000 individuals with Spina Bifida and help prevent future cases of this birth defect.

While many in the Spina Bifida community do participate in health policy advocacy efforts, still too many mistakenly believe that it is not for them. Examples given to us as to why people do not participate include: a dislike of politics, thinking that communicating with elected officials takes a lot of time and/or

*Corresponding author.
©2007 with author.
Published by TheScientificWorld: www.thescientificworld.com does not make a difference, feeling that an expertise in political science or civics is necessary, and not wanting to "bother the Senator with what I think." Given these concerns, it is critical to remember that we all employ our Members of Congress – our tax dollars pay for their salaries, health insurance, travel to/from Washington, retirement plans, etc. As such, we all have every right to express our opinions to them and ask them to take action on our priority concerns. In fact, the Constitutions grants each citizen the "right to petition Congress for redress of grievances." Asking elected officials to support the Spina Bifida community is merely exercising our Constitutional right.

Affected individuals and family members, physicians, nurses, and other caregivers inherently are advocates – every day they take advocacy action on their own behalf or on behalf of individuals with Spina Bifida (e.g. urging that a health plan provide coverage for a needed service). Advocacy is merely voicing your opinion (by any number of ways – email, letter, phone call, fax, or an in person meeting) on an issue to an elected official and asking that action be taken to address your concern. All health policy advocacy requires is taking this skill and applying it in a slightly different arena. Individuals with Spina Bifida and their family members and caregivers know first-hand the health care, vocational, educational, and other resources needed to maximize quality of life for someone with this birth defect – that makes all of these people a credible, trusted, and valuable voice in public policy considerations. No special knowledge of civics is necessary – the first-hand experience of the impact of Spina Bifida will speak for itself.

Members of Congress are most responsive to people from their own states and communities, and to spur them to action, they must hear from Spina Bifida community members about their concerns. Congressional staff report that all communications to a Member of Congress are counted so emails, phone calls, faxes, and letters do make a difference. The most valued communication is a personalized letter or an in-person visit – which does take a little more time. As such, SBA works to initiate, coordinate, and follow-up on contact from constituents to their elected officials.

### Grassroots Advocacy

The delivery of coordinated broad-based messages by individuals at the local level to elected officials generally is referred to as a "grassroots effort." SBA makes taking action very easy – through its online Advocacy Center (www.sbaa.org) – Spina Bifida community advocates have access to all the information they need (e.g. tips for calling, emailing, or meeting with elected officials, template letters and talking points for phone calls to Congressional offices, etc.). SBA supports and encourages "grassroots" advocacy by posting background and current information on its web-based Advocacy Center, issuing "action alerts" to its members when communication with Congress will help influence a particular decision affecting the Spina Bifida community, and including regular articles in its newsletter, Insights, updating members on advocacy efforts and encouraging them to take action to support SBA's public policy agenda. By sending an email through the SBA Advocacy Center, individuals can educate Members of Congress and staff about what actions are necessary to support people with Spina Bifida.

### Grasstops Advocacy

While "grassroots" advocacy is effective and important, increasingly Members of Congress and their staff are becoming overwhelmed by emails, letters, faxes, and phone calls from constituents contacting them about thousands of issues of concerns. As such, it can be difficult to garner support and attention from elected officials for an issue like Spina Bifida that is not as recognizable or understood as other health concerns like cancer, diabetes, or heart disease. Moreover, while all Members of Congress are important and need to hear from constituents in the Spina Bifida community, certain Members sit on committees which have jurisdiction over issues of priority to SBA and therefore they are in a position daily to have a direct influence on funding and programmatic issues impacting the Spina Bifida community.

As such, SBA also engages in what we refer to as “grasstops” advocacy – a term that connotes a small group of constituents who live in certain targeted states and/or Congressional Districts of Members of Congress. SBA’s “grasstops” approach involves identifying, training, and mobilizing influential Spina Bifida community leaders in targeted communities who can reach elected officials at the federal level with facts and stories tailored to their state’s/district’s interests. The Members of Congress that SBA targets for particular “grasstops” advocacy and outreach sit on the Senate Appropriations Committee and its Labor-Health and Human Services-Education (LHHS) Appropriations Subcommittee, the Senate Health, Education, Labor, and Pensions (HELP) Committee, the Senate Finance Committee, the House Appropriations Committee and its Labor-Health and Human Services-Education (LHHS) Appropriations Subcommittee, the House Energy and Commerce Committee and its Health Subcommittee, and the House Ways and Means Committee and its Health Subcommittee (for an explanation of each committee’s jurisdiction, please see Table I).

SBA has identified a small and growing group of “grasstops” advocates who engage in efforts both at home as well as in Washington, DC. These individuals have developed close working relationships with local and Washington-based Congressional staff as well as the elected leaders themselves – educating them about Spina Bifida, explaining how the National Spina Bifida Program and other federal efforts are helping to improve quality of life for people with Spina Bifida, and encouraging them to support increased funding for these important initiatives. For example, the Branson family of Madison, Mississippi, travels to Washington, DC once a year to visit with the Mississippi Congressional delegation, which includes the senior most Republican in the Senate on the Appropriations Committee, a senior Republican member of the Senate Finance Committee, a senior Republican member of the House LHHS Appropriations Subcommittee, and an up-and-coming Republican member of the House Energy and Commerce Committee. While on Capitol Hill for their annual visit, Abigail Branson, a nine-year-old with Spina Bifida, and her mother Susan, visit with Members of Congress and their staff to discuss with them the health, educational, and social challenges of living with Spina Bifida and the benefits and assistance provided vis-à-vis the information and resources developed and disseminated by the National Spina Bifida Program. Throughout the course of each year, before and after their Washington trips, to maintain strong relationships and keep elected officials up-to-date on issues impacting the Spina Bifida community, Abigail and Susan visit policymakers and their staff at home in Mississippi and call and write the Members and staffers in Washington. All of these activities constitute “grasstops” advocacy. This model is replicated throughout the United States with SBA grasstops advocates in other key states and Congressional districts reaching out to and developing relationships with Members of Congress and their staff to expand the base of Congressional support for the Spina Bifida community.

### *Washington Based Advocacy*

SBA staff and volunteer leaders regularly visit Capitol Hill to meet with Members of Congress and their staff to educate them about Spina Bifida, ask them to take action to support the community, and answer concerns and inquiries. Throughout the course of a year, SBA advocates typically visit more than 60 Congressional offices, principally those Members who sit on the key committees described above. In addition, SBA regularly corresponds with Members of Congress – sending letters and submitting testimony – regarding the funding and policy needs of the Spina Bifida community for the coming year. Also, it is not uncommon for Congressional staff to contact SBA with questions about Spina Bifida or for Congressional offices to ask SBA to help them respond to constituent inquiries regarding Spina Bifida research, treatment, or prevention efforts.

### Congressional Spina Bifida Caucus

In existence for approximately four years, the Congressional Spina Bifida Caucus was formed by Representatives Chris Smith (R-NJ) and Bart Stupak (D-MI) – who serve as its co-chairs – to help elevate awareness among Members of Congress of Spina Bifida, the challenges faced by the 70,000 affected individuals, and the interventions, programs, and policies that could help reduce and prevent suffering from this birth defect. Currently, nearly 50 Members of the U.S. House of Representatives participate in this important bipartisan group. Any Member of Congress can join and the only requirement is an interest in working on a bipartisan basis to improve quality of life for people with Spina Bifida and to support efforts to prevent future Spina Bifida affected pregnancies. The Caucus has hosted a number of Congressional briefings, written to federal agencies on behalf of SBA urging action in a number of areas for the Spina Bifida community, and sponsored “Dear Colleague” letters – which are letters circulated to Members of Congress for their signature, asking for fellow Members’ support for a particular priority. Each year, the co-chairs of the National Spina Bifida Caucus, along with other Spina Bifida community champions, such as Representatives Dan Burton (R-IN), Sherrod Brown (D-OH)[1], and Ileana Ros-Lehtinen (R-FL), circulate an appropriations “Dear Colleague” letter, requesting Members show support by signing on a joint letter to Appropriations Committee leaders asking for increased funding for the National Spina Bifida Program. These letters usually garner dozens of signatures and help illustrate to the Members of the House Appropriations Committee that a broad constituency of their colleagues support boosting funding for this important program.

### Congressional Briefings

SBA has hosted numerous briefings on Capitol Hill to educate Congressional staff and others in the Washington, DC health care community about Spina Bifida and the needs of individuals living with this birth defect. Most recently, in May 2006, SBA co-hosted a briefing with the National Council of La Raza to present a Congressional Briefing entitled “Steps the U.S. Can Take to Improve Quality-of-Life and Reduce Health Disparities Associated with Spina Bifida.” Despite an overall reduction in the number of affected pregnancies, CDC reports that the rate of Spina Bifida in the Hispanic population is almost seven in 10,000 births, nearly 40 percent higher than the non-Hispanic rate. As such, the briefing focused on the need to address this dramatic health disparity through increased health education and awareness efforts and enrichment of corn products imported into the United States, which are found in many foods consumed by the Hispanic community. Briefing speakers included: Dr. José Cordero, Director of the CDC National Center on Birth Defects and Developmental Disabilities, Emily Gonzalez-Abreu, the mother of a child with Spina Bifida, Charles Kamasaki, Senior Vice President of the National Council of La Raza, and Hal Pote, President of the Spina Bifida Foundation. Events such as this help to increase Congressional understanding and appreciation of the myriad factors impacting Spina Bifida incidence and morbidity and provide an important platform for SBA to deliver its message directly to the policymakers in a position to make a difference for people with Spina Bifida.

### Federal Agency Advocacy

While much of SBA’s public policy advocacy efforts are focused on the actions of Congress, SBA maintains a contemporaneous focus on the activities of relevant federal agencies, including, CDC, the Agency for Healthcare Research and Quality (AHRQ), the National Institutes of Health (NIH) and its myriad subinstitutes, and the Department of Veterans Affairs which operates a Spina Bifida Health Care Program – for the natural children of service members who served in Korea and Viet Nam from September 1, 1967 through August 31, 1971 and who where exposed to Agent Orange. Generally, SBA’s advocacy efforts with federal agencies and programs are collaborative, with SBA working with federal agency staff to ensure that the programs and initiatives related to Spina Bifida research, care, and benefits are being operated and delivered in alignment with Congressional intent. For example, once a year, leaders from the SBA and the Spina Bifida Foundation travel to Atlanta to meet with staff of the CDC National Spina Bifida Program to review the program's work to-date, discuss plans for the coming year, and dialogue on any pending issues, concerns, and questions.

The Spina Bifida Association works collaboratively with several federal agencies. At present, SBA is working with the Agency for Healthcare Research and Quality and its HHS sister agency, CDC to lay the foundation for the National Spina Bifida Patient Registry. Building on the relationship with AHRQ and the CDC and with funding fueled by advocacy efforts, a patient registry is in process. It is our belief that such a registry could lead to a revolution in the care of people with Spina Bifida and solve the growing problem of a lack of treatment options for adults living with this devastating birth defect. Further, we believe that the complexity of care for Spina Bifida could serve as a blueprint for many living with chronic disease.

The Spina Bifida Association has partnered with the National Institute for Child Health and Human Development to help recruit for its landmark study, Management of Myelomeningocele Study (MOMS). This important study is a randomized clinical trial evaluating the effects of fetal surgery. The Spina Bifida Association wrote an article that appeared in a publication of the American College of Obstetrics and Gynecology to publicize the study to physicians whose patients could be appropriate for the study. An increase in the number of study participants occurred as a result of the article.

The Spina Bifida Association, the CDC and the Veterans Administration have also worked together to examine the database of patients who received care under the Agent Orange program. The review of the database is providing answers to the widely varied health, educational and vocational needs of people with Spina Bifida.

### Corporate Responsibility

The Spina Bifida Association works with corporations to help to meet its goals. Corporate responsibility and leadership to improve the world is part of our American culture. In fact, during the last decade, corporations have taken an increasingly more prominent role in charitable giving. Of the $260.28 billion given to nonprofit organizations in the United States in 2005, corporations gave about 5.3% or $13.77 billion. Corporate giving steadily has increased from $5% in 2004 and from 4.5% in 2003. Recently, *The Wall Street Journal* conducted a study of more then 200 financial executives who received bonuses in 2006 of $2 million or more in cash. The bankers and traders involved in this study reported that they plan to give about 4 percent of their bonuses to charity. This makes a good case for increasing efforts to secure corporate contributions as well as strategically target corporate executives for individual gifts.

The Spina Bifida Association and the Spina Bifida Foundation have mobilized our corporate interest to help advance our dual mission to improve the quality of life for those with Spina Bifida and their families as well as to work to prevent this serious birth defect. First, through our annual meeting each June, we have built good relations with many companies that supply products to improve the quality of life and serve the needs of those with Spina Bifida and their families. Second, each October, we develop corporate sponsorships that provide recognition to these corporate donors as well as generate awareness for the prevention of Spina Bifida through our annual Gala.

In the same way as corporations, individuals have increasingly looked for ways to make the world a better place by giving more to charities. Spina Bifida Association and Spina Bifida Foundation are building the foundation for the future of Spina Bifida research through individual giving opportunities, which have recently translated into two new endowments, and another unrestricted gift that will cover part of our cost of operations.

**Table I.**
**Committees of the U.S. Congress with Jurisdictions of Relevance and Priority to the Spina Bifida Association**

| Senate Committees | House Committees |
|---|---|
| **Senate Appropriations Committee**<br>Has jurisdiction over all federal spending | **House Appropriations Committee**<br>Has jurisdiction over all federal spending |
| **Senate LHHS Appropriations Subcommittee**<br>Has jurisdiction over federal spending for AHRQ, CDC, National Spina Bifida Program, and NIH | **House LHHS Appropriations Subcommittee**<br>Has jurisdiction over federal spending for AHRQ, CDC, National Spina Bifida Program, and NIH |
| **Senate HELP Committee**<br>Has jurisdiction over policy and programmatic issues related to AHRQ, CDC, National Spina Bifida Program, and NIH | **House Energy and Commerce Committee**<br>Has jurisdiction over policy and programmatic issues related to AHRQ, CDC, Medicaid, National Spina Bifida Program, and NIH |
| **Senate Finance Committee**<br>Has jurisdiction over policy and programmatic issues related to Medicare, Medicaid, Social Security Disability Insurance, and Supplemental Security Income | **House Ways and Means Committee**<br>Has jurisdiction over policy and programmatic issues related to Medicare, Social Security Disability Insurance, and Supplemental Security Income |

To learn who represents you in Washington and whether either of your Senators or your Representative sit on any of these important committees, visit: www.senate.gov or www.house.gov.

### FOOTNOTE

1. Representative Sherrod Brown was elected to serve in the U.S. Senate on November 7, 2006. As such, he will be Senator Sherrod Brown beginning January 2007 and will serve in that capacity for a full six-year term.

**This article should be cited as follows:**

Brownstein, C., Paul, I.H., and Quinn, A.H. (2007) Spina Bifida Advocacy – A Case Study In Public Policy Success *TheScientificWorldJOURNAL:* TSW Urology **7**, 1538–1543. DOI 10.1100/tsw.2007.194.
